# Hearing spiritually significant voices: A phenomenological survey and taxonomy

**DOI:** 10.1136/medhum-2020-012021

**Published:** 2020-12-07

**Authors:** Christopher C H Cook, Adam Powell, Ben Alderson-Day, Angela Woods

**Affiliations:** 1 Theology and Religion, Durham University, Durham, UK; 2 Psychology, Durham University, Durham, Durham, UK; 3 Institute for Medical Humanities, Durham University, Durham, UK

**Keywords:** Medical humanities, psychiatry, theology

## Abstract

Whereas previous research in the medical humanities has tended to neglect theology and religious studies, these disciplines sometimes have a very important contribution to make. The hearing of spiritually significant voices provides a case in point. The context, content and identity of these voices, all of which have typically not been seen as important in the assessment of auditory–verbal hallucinations (AVHs) within psychiatry, are key to understanding their spiritual significance. A taxonomy of spiritually significant voices is proposed, which takes into account frequency, context, affect and identity of the voice. In a predominantly Christian sample of 58 people who reported having heard spiritually significant voices, most began in adult life and were infrequent experiences. Almost 90% reported that the voice was divine in identity and approximately one-third were heard in the context of prayer. The phenomenological characteristics of these voices were different from those in previous studies of voice hearing (AVHs). Most comprised a single voice; half were auditory; and a quarter were more thought-like (the rest being a mixture). Only half were characterful, and one-third included commands or prompts. The voices were experienced positively and as meaningful. The survey has implications for both clinical and pastoral work. The phenomenology of spiritually significant voices may be confused with that of psychopathology, thus potentially leading to misdiagnosis of normal religious experiences. The finding of meaning in content and context may be important in voice hearing more widely, and especially in coping with negative or distressing voices.

## Introduction

In addressing the ‘intersections, exchanges and entanglements between the biomedical sciences, the arts and humanities, and the social sciences’,1 the medical humanities should in theory involve a significant contribution from theology and religious studies. These disciplines have a distinct contribution to make to deepening understanding of both illness and wider human experience. Christian theology, for example, is something that people ‘do’ and is not merely an academic exercise; it is contextual, rooted in the specifics of time, place and culture; it is inclusive of the beliefs, thinking and expectations of ordinary Christians and is not limited only to the seminary or university.2 Theology and religious studies are concerned with both the beliefs and unbeliefs within which people make sense of their experiences. Yet, with some notable exceptions,3 in practice, such contributions are often lacking.4

The present paper seeks to remedy this omission in relation to the phenomenology of voice hearing. Voice hearing provides a particularly rich and interesting case in point, with a large and growing literature involving the biomedical and social sciences, as well as contributions from virtually all disciplines within the humanities.5 Contributions to this literature from theology and religious studies have, however, tended to be published outside the medical humanities framework.6 Medical humanities analyses have a part to play in changing debates in psychiatry and the related sciences about such matters as the proposed phenomenological continuum of voice-hearing experiences. They address, for example, the thorny questions of methods appropriate to conceptualising, testing and investigating that continuum and the contexts in which this is done. They contribute to a critical approach to employment and testing of taxonomies of voice-hearing experiences.

The empirical research presented in the present paper provides an example of work within this wider context—work which is poised to speak not only to phenomenological and clinical studies but also to anthropological studies,7 philosophy of psychiatry publications8 and first-person accounts.9 We seek to attend to first-person accounts (as much as possible on their own terms but also using a standardised instrument for data collection) and so identify new categories/taxonomies, as well as considering how these could be fed back to other phenomenological/clinical contexts.

### Spiritual and religious voices

Research conducted over the last 10 years and more has shown that voices (auditory–verbal hallucinations (AVHs)) are experienced widely in the general population and are not necessarily indicative of psychopathology. People who hear voices who are diagnosed with mental disorders are typically found to have more negative emotional experiences in association with their voices and more maladaptive coping mechanisms.10 Others, in contrast, experience their voices positively, describe having more control over their experiences and may interpret them in a spiritual or religious context.11 Comparison studies have often used non-clinical control groups selected for frequent experiences of voice hearing, but not all such experiences are frequent. Selection on this basis biases against certain types of voices, including infrequent spiritually significant voices, and thus may skew results towards finding an apparent phenomenological overlap.

Historically, experiences of hearing voices have been both theologically and spiritually highly significant.12 In contrast, psychiatry has a history of interpreting religious voices negatively.13 Recent research14 has proposed placing non-clinical voice-hearing experiences on a continuum with those associated with psychosis and, at least potentially, this may be destigmatising for those who suffer from psychosis. However, phenomenological continuity does not necessarily imply continuity of underlying mechanisms.15 Luhrmann16 has shown that religious voices are phenomenologically distinct from the voices reported in psychosis and that those who hear religious voices occupy social contexts within which such voices are affirmed and expected, and are engaged in spiritual practices which cultivate the inner senses. Furthermore, those within such religious settings who hear these voices are distinguished from those who do not by their psychological proclivity to ‘absorption’. Whereas for those with psychosis the continuum hypothesis may be destigmatising, for those who hear spiritually significant voices, it may in fact have the opposite effect and risk pathologisation of essentially normal and socially integrated human experiences.

Among positive contemporary experiences of religious voices are those of some Christians who hear voices in the context of prayer, typically identified as the voice of God.17 While such voices do share a surprising degree of phenomenological similarity to those of other groups of voice hearers, they are interpreted very differently. In terms of social identity, such experiences are understood by those who have them neither as ‘voice hearing’18 nor as evidence of mental disorder. On the contrary, such experiences are often affirmed within church communities where they may be understood as evidence of good spiritual health.

Similar observations have been made with respect to other religious groups. For example, spiritualist mediums have been shown to score similarly on clinical measures that are intended to measure psychotic symptomatology19 and to report similar phenomena as voice hearers with a psychosis diagnosis. However, mediums not only report higher levels of control and overtly positive emotional valence associated with their voices, but also tend to hear voices within and to receive socialised interpretations from their churches, ‘circles’, and clients which value and legitimise the voices.20 Both Christians who hear voices and spiritualist mediums who report clairaudience have been shown to seek voice-hearing experiences through a combination of group learning and individual practice.21

To date, there has been very little systematic comparison of the voices that Christians hear, such as those studied by Luhrmann and others, with AVHs—most work has been anecdotal, based on small numbers of case studies, or ethnographic. However, comparing the phenomenology of such voices is no mean feat. Reticence about appearing to imply that such voices are pathological may play a part here, but there are also methodological difficulties. AVHs are very varied, clinical scales can lack nuance, and existing research tools could easily miss aspects of experience specific to Christians and other spiritual groups. Open-ended tools are generally preferable, capturing people’s voices more in their own words.

A recent successful example of research conducted in this vein is that of Woods *et al*,22 who recruited 153 participants from clinical networks, hearing voices groups and other mental health forums. Their survey showed that people who hear voices typically hear multiple voices and describe their voices as characterful. Less than half describe their voices as literally auditory, with most describing them as either thought-like, or else a mixture of thought-like and out loud. Two thirds experienced bodily sensations in association with their voices, and these sensations were significantly associated with abusive or violent voices. Voices were associated with a variety of emotional states, including, in about one-third, positive emotions. Only 29% of participants in the study by Woods *et al* self-identified as ‘Christian’, although the majority saw themselves as spiritual or religious (only 29% said that they had no religious beliefs).

Employing a similar methodology to Woods *et al*, we sought to compare their findings with those obtained from a largely Christian sample invited to report specifically on ‘spiritually significant’ voices. The aim was to draw a phenomenological comparison and to better understand how Christians (and others) interpret voices that they understand to be spiritually significant.

### Spiritually significant voices

Voices are significant to many—probably most—people who hear them. However, significance may take a variety of forms. Quite apart from spiritual significance, voices may be personally significant (eg, due to association with painful memories or life experiences); significant for the future (eg, due to pessimism about the possibility of getting rid of distressing voices); or they may be taken as significant evidence of a particular diagnosis (including of mental disorder). 23 They might be significant as a source of creativity.24 They may be experienced as socially and personally significant, conferring a particular sense of self-identity as a voice hearer and providing a source of meaning.25 Spiritually significant voices may or may not be significant for any or all of these reasons, but they are distinctive by virtue of the spiritual interpretation that is placed on them or—perhaps more correctly—is an integral part of the experienced voice.

In this study, we have left our subjects to self-identify their voices as spiritually significant. Spiritual significance might potentially be understood in terms of the context, content, identity or form of the voice, and as something perceived or assigned by the person hearing the voice, or their faith community or both. At the outset, we left this open to our participants to decide.

Spirituality is a contested term which is difficult to define. It may or may not be understood as associated with religion; it may be understood as the antithesis to religion. It is often understood as concerned with important relationships—with self, others or a transcendent order. It is seen as closely linked to meaning and purpose in life.26 Again, we did not impose any definition of spirituality at the outset, but given the mode of ascertainment of the subjects (below), we may assume that many would have interpreted the concept in traditionally Christian theological terms.

## Methodology

Our study design employed both quantitative and qualitative methodologies.

### Participants

Our questionnaire was made available online from June 2018 to August 2019. The primary point of access to this was via the *Church Times* website, where a link was made available in connection with three articles published (in print and online) by one of us in 2017.27 An advertisement was also placed in the print copy of the *Church Times*, and attention was drawn to the survey by word of mouth. The *Church Times* provides independent reporting of news relating to the Christian Church and the wider world. Weekly distribution of the print copy is 20 000, and the website attracts 26 000 unique visitors each week. While consideration was given to publicising the survey in other religious periodicals, we hoped to gain a more homogenous subject sample by limiting the survey primarily to *Church Times* readers.

A large number of readers of the *Church Times* also wrote emails and letters in response to the *Church Times* articles, and some of their stories are published, in their own words, elsewhere.28 Correspondents were all invited to take part in the survey described in the present paper, but no check was (or could be) made on whether or not they did. The survey described here was completely anonymous.

Readers of the *Church Times* and others were invited to take part in the survey if they had at any point in their lives heard ‘spiritually significant voices’. The invitation stated:

Some people find the term ‘hearing voices’ a useful way to describe their experience; others refer more specifically to hearing God, angels, saints, demons or other spiritual beings. Others hear sounds but not speech, describe loud thoughts or feel the presence of God, angels, demons or other beings. For some people, such experiences have been rare—perhaps once in a lifetime—and for others, they are ongoing. Our aim is to develop a better understanding of all of these experiences. Anyone can take part who has had first-hand experience of hearing a spiritually significant voice, as long as they are 16 years of age or over.

The question of whether or not the voices of interest were auditory (‘out loud’) was deliberately not mentioned.

### Patient and public involvement

Neither patients nor public were directly involved in the design or conduct of the research in any way. Research questions were informed by the engagement of our research team with voice hearers over a number of years. Findings will be disseminated through the Understanding Voices website, which has been produced in close collaboration with voice hearers, their families and allies, as well as with mental health professionals.

### Questionnaire

The questionnaire was based on that used by Woods *et al*, with minor revisions to make it more accessible for the intended audience. In particular, references to hearing voices were replaced with ‘hearing spiritually significant voices’. However, participants were also asked whether they had ever heard ‘non-spiritually significant voices’. As with the original instrument, questions were designed to be non-leading and unbiased, with a mixture of close-ended and open-ended questions. Participants were provided with free-text boxes to describe the phenomenology of their voice-hearing experiences. Additional questions, not in the original instrument, included ‘Who do you believe is speaking to you?’ and ‘What has this experience (or experiences) meant to you?’ All the core questions concerning the characteristics of voices were retained in order to enable direct comparison with the original study:

- Please try to describe your spiritually significant voices(s) and/or voice-like experiences.
- How, if at all, are these experiences different from your own thoughts?
- How often have you had such experiences?
- How, if at all, are these experiences different from hearing the voice of someone who is present in the room?
- Does it feel as though the voices that you hear have their own character or personality?
- How old were you when you first had such an experience? Can you tell us a bit about what life was like for you, and how you were feeling, when you first started hearing (the) voice(s) or having voice-like experiences?
- What kinds of moods or emotions are associated with your voice(s)?
- Does your body feel different when you experience the voice(s)?
- Do you know when you are about to experience a voice? If so, how?
- How, if at all, do your voices affect your relations with other people?
- What other kinds of experience, if any, accompany your experiences of voices?
- Are there any aspects of your experience of hearing voices that this questionnaire has not covered? If so, please tell us about them here.

### Data analysis

Characteristics of the voices heard were analysed using the same phenomenological categories and themes as Woods *et al*. For example, qualitative responses were studied for the presence of ‘conversational’, ‘characterful’, ‘abusive’, ‘auditory only’, ‘thought-like only’ and ‘mixed’ voices. For most categories not all participants provided enough information to enable a confident allocation (and percentages therefore do not add up to 100%). However, in several cases—for example, when assessing characterful voices or ‘bodily sensations’ accompanying voices—categorisation was based on answers to single, specific questions addressing these issues. By applying Woods *et al*’s analytical frame, we were able to compare our data directly with theirs.

Decimal fractions were rounded up/down and so statistics presented in the results do not always total exactly 100%.

### Taxonomy of spiritually significant voices

We further classified the voices of our respondents according to an eightfold taxonomy of different kinds of Christian voice-hearing experiences developed by Cook.29 This provisional taxonomy, arising from research on first person Christian accounts of hearing spiritually significant voices, is based primarily on the content of what the voices say, but also takes into account affective experience, life context, frequency/timing of voice-hearing experiences and the quality/identity of the voice. The categories are not mutually exclusive and some people may have more than one kind of experience. They are not jointly comprehensive. However, they do serve to map out some of the more significant varieties of spiritually significant voice experiences. Notably, some voices are remarkable by virtue of being a once in a lifetime experience, or at least very rare, whereas others seem to be a frequent, if not daily, experience. Some are positively affectively laden, and others are negative and distressing. The taxonomy emphasises meaning, and so a voice conveying identical words might be classified differently in different contexts, or where there is a differing affective tone. The eight categories are summarised in table 1.

**Table 1:** Taxonomy of Christian voice-hearing experiences (after Cook 2020)

Category	Frequency	Context	Affect	Identity
Conversion	Once in a lifetime/infrequent	Conversion experience	Positive	Usually understood to be God (or Jesus or the Holy Spirit)
Calling		Vocational decision		
Crisis		Life/death crises		
Comfort		Difficult circumstances		
Confirming/clarifying		Uncertainty		
Communications	May be infrequent or ongoing	Wider relevance	May be positive or negative	May be God, another personal spiritual being (good or evil), or an alter ego
Conversational		Inner dialogue		
	Usually more frequent/everyday	Prayer		
Companions		Daily life		

Voices classified as ‘conversion’ and ‘crisis’ are associated temporally with significant events—for example, becoming a Christian or a life-threatening emergency. Voices categorised as ‘calling’ are associated with particular vocational decisions — for example, to become a priest. Comforting, confirming and clarifying voices are typically also infrequent (although perhaps not ‘once in a lifetime’) and associated with particular life circumstances, typically having a positive affective tone at a time of emotionally unsettling or difficult experiences.

In one sense, all spiritually significant voices are ‘communications’. However, some voices seem to offer communications about particular matters quite apart from the contextual concerns of the preceding categories in table 1 and have wider significance. Neither are they necessarily examples of the more frequent experiences of voice hearing typical of companion voices or conversational voices. In this taxonomy, it is the criterion of wider relevance that marks out these voices as different and distinctive. For example, they may convey a message for another person, or about other people, or about the human condition. This is not a miscellaneous or ‘other’ category, and voices were only included here where the criterion of wider relevance was met.

Some voices have a particularly marked sense of social agency and/or ‘presence’. In this sense, particularly when they are frequent or enduring experiences, they become companions. Companion voices may be benevolent or hostile. In one case reported by Cook,30 the companion voice was complex in terms of its relationship with mental health, faith and life context. It might generally be termed ‘evil’ or ‘negative’, but it also occupied a complex inner space alongside that of the voice of God.

While companion voices might also be described as conversational, some conversational voices seem not to be identifiable as companions. Prayer is not uncommonly understood as conversation in Christian circles, for example, as illustrated in Luhrmann’s book *When God Talks Back*.31 We might say that in this case God is the ‘companion’, but the voice of God is not typically experienced outside the context of prayer and is not identifiable as a companion in quite the same way as some other voices are. Companion voices, as defined here, operate outside the specific context of prayer and are more associated with a sense of presence. (The difference here is subtle and is more about phenomenology than theology.)

## Results

A total of 58 participants completed our survey, with all participants reporting having had a voice-hearing experience on at least one occasion. No responses were excluded. The mean number of words submitted per person, in response to all free-text questions combined, was 457.0 (SD 430.4, minimum 41, maximum 2713).

More than two-thirds of participants were female and the majority (86%) were 45 years old or older (table 2). Seventy-nine per cent of respondents either reported having never received a psychiatric diagnosis or else gave no response. Among those who did report a diagnosis, the most commonly reported conditions were depression (n=5) and post-traumatic stress disorder (n=3) (see table 2).

**Table 2:** Demographic and diagnostic information

	n=58
Age (years)	
0–18	0
19–24	2 (3%)
25–34	3 (5%)
35–44	3 (5%)
45–54	5 (9%)
55–64	11 (19%)
65–74	19 (33%)
75–84	13 (22%)
85+	2 (3%)
Gender	
Male	16 (28%)
Female	41 (71%)
Other	0
No answer	1 (2%)
Country of residence	
UK	56 (97%)
USA	2 (3%)
Country of origin	
UK	51 (88%)
USA	2 (3%)
Mauritius	1 (2%)
No answer	4 (7%)
Religious affiliation	
Christian (general)	19 (33%)
Christian (Anglican)	32 (55%)
Christian (Quaker)	2 (3%)
Christian (Methodist)	1 (2%)
Hindu (Vedanta)	1 (2%)
Jewish	1 (2%)
Pagan/Druid	1 (2%)
Agnostic	1 (2%)
Psychiatric diagnosis	
No diagnosis	44 (76%)
Depression	5 (9%)
Post-traumatic stress disorder	3 (5%)
Addiction	1 (2%)
Eating disorder	1 (2%)
Generalised anxiety disorder	1 (2%)
Panic disorder	1 (2%)
Schizophrenia	1 (2%)
Bipolar disorder	0
Dissociative identity disorder	0
Obsessive–compulsive disorder	0
Schizoaffective disorder	0
No answer	2 (3%)

More than half of participants reported voice hearing for the first time at age 21 years old or older. The circumstances surrounding onset were unclear, with exactly 50% of participants failing to provide adequate information on this matter (table 3). Seven (12%) of our participants recalled initial voice-hearing experiences coinciding with moments of crisis, indecision, career change or other transitional events, but without indicating clearly whether or not these were positive, negative, traumatic or substance-induced circumstances. Thus, we created an additional category for ‘transitional’ onset to supplement those originally used by Woods *et al.*

**Table 3:** Onset, frequency and predictability of voices

	Present study (n=58)	Woods *et al* 2015 (n=153)
**Voice onset**		
Childhood (0–11)	6 (10%)	52 (34%)
Adolescence(12–20)	11 (19%)	32 (21%)
Adulthood (21+)	32 (55%)	29 (19%)
Uncertain	9 (16%)	40 (26%)
Circumstances		
Positive	4 (7%)	17 (11%)
Negative	15 (26%)	36 (24%)
Traumatic	2 (3%)	35 (23%)
Transitional	7 (12%)	
Substance use	1 (2%)	10 (7%)
Not enough information to classify	29 (50%)	55 (36%)
Frequency (lifetime number of occurrences)		
1	15 (26%)	
2–4	12 (21%)	
5–10	4 (7%)	
11+	7 (12%)	
Uncertain (but more than 1)	20 (35%)	
Prediction		
Can predict/anticipate	1 (2%)	67 (44%)
Cannot predict/anticipate	57 (98%)	70 (46%)
Continuous voices	0 (0%)	22 (14%)

For most respondents, the hearing of a voice had been a relatively infrequent experience, occurring ten times or less over the course of a lifetime (table 3). For a quarter, it had been a once in a lifetime experience. For a minority (12%) it had occurred on eleven or more occasions and, although we had no category to measure this, we may speculate that for some of these it may have been an ongoing and frequent experience, although perhaps not “continuous” in the sense reported by 14% of respondents in the study by Woods *et al*. All but one (98%) claimed not to be able to predict the experience. A small subset of six participants, however, did provide responses suggesting that they could increase the likelihood of hearing a divine voice by entering into specific contexts, such as a ‘worship service’ or a state of ‘prayer’.

Approximately half of participants reported only having auditory voices (table 4). The remainder were fairly evenly split between either experiencing only thought-like voices or experiencing a combination of both. Approximately half of our participants felt that the voices originated internally, with the other half reporting external voices.

**Table 4:** Characteristics and identity of voices

	Present study	Woods *et al* 2015
**Characteristics**	(**n=58**)	(**n=153**)
Auditory only	30 (52%)	67 (44%)
Thought-like only	14 (24%)	14 (9%)
Mixed auditory and thought-like	12 (21%)	56 (37%)
External	28 (48%)	69 (45%)
Internal	30 (52%)	67 (44%)
Single voice	50 (86%)	10 (7%)
More than one voice	8 (14%)	124 (81%)
Bodily sensations	15 (26%)	101 (66%)
Characterful	29 (50%)	106 (69%)
Commands	20 (34%)	8 (5%)
Conversational	6 (10%)	57 (37%)
Abusive	5 (9%)	54 (35%)
**Identity of the voice†**	(**n=58**)	(n=24)*
God	46 (79%)	5 (21%)
Holy Spirit	7 (12%)	0 (0%)
Jesus/Christ	7 (12%)	1 (4%)
Angel/messenger	6 (10%)	3 (13%)
Self (eg, ‘deepest, non-ego self’)	3 (5%)	3 (13%)
God–other (creator/Lord)	2 (3%)	0 (0%)
Devil/demons	2 (2%)	7 (29%)
Goddess	1 (2%)	0 (0%)
Spirit guide	1 (2%)	3 (13%)
Other	5 (9%)	13 (54%)
Unknown	11 (19%)	0 (0%)

*Subjects reporting spiritual/supernatural voice identity

†Some participants had multiple voices/speakers and some gave multiple attributions. Percentages therefore sum to more than 100%.

The majority of our participants (86%) recalled only hearing a single voice (table 4), although that same voice may have recurred on separate occasions. This contrasts with the study by Woods *et al*, in which 81% heard more than one voice. Two respondents appeared to distinguish between different persons of the Trinity, although it was not entirely clear that the voices were different. For example, one said ‘God was just a felt presence but Jesus has been visible’. Given the complexity of Christian theology of the Trinity, we have treated these here as single voices.

Most voices were not accompanied by bodily sensations (table 4). Among those who did report bodily sensations, these sensations included ‘touch’, ‘warmth’, ‘a physical pull’ or even a ‘trance state’ (box 1).

Box 1 **Bodily sensations accompanying voices**

‘I have felt different sensations of touch, being flooded with warmth…’

‘From time to time I have felt a warm glow, or a physical pull…’

‘It felt that [my body] was ceasing to function’

‘Physical exhaustion’

‘Trance state…energy leaving me’

‘I get heavy hands, or I feel that someone is holding my hands’

‘I can feel a visceral reaction in the gut or tug behind the ribcage’

‘It feels sometimes lighter but sometimes heavier’. ‘Sometimes I will get heart palpitations’

‘…in a state more or less disconnected from the body’

‘I get goosebumps. Most strongly/commonly on my shoulders and upper back’

Voices in our study, as compared with Woods *et al*, were less likely to be characterful, more likely to give commands, and less likely to be conversational (table 4). Only five (9%) participants reported violent or abusive voices (table 4) and, of those, only three associated the voices with negative emotions.

Almost four-fifths identified the voice that they heard as God’s voice. A significant minority also identified the voice as Jesus and/or the Holy Spirit. When taking into account all forms of reference to God, 51 respondents (88%) identified the voice as divine. Only two identified demonic voices. This represents a very different profile than that of respondents in the survey by Woods *et al*. Among the 24 subjects in that study who identified their voices as spiritual, only 5 (21%) identified the voice as that of God, but 7 (29%) identified their voices as demonic (see table 4). Among these 24 respondents, there was much greater religious diversity (only six identified as practising Christians, with a further four as former/non-practising Christians), and only 3 had not received a psychiatric diagnosis.

Participants, on the whole, reported the significance of their voices in highly favourable terms (box 2). Only eight (14%) participants reported hearing voices that were ‘not spiritually significant’.32 Qualitative responses indicate that these included an alcohol-induced voice, a screaming woman coinciding with menopausal hot flushes, hypnagogic voices and one ‘radio-like’ conversation heard coming from the next room. For the majority who only heard spiritually significant voices, free-text responses describing any ‘moods or emotions’ accompanying the voices as well as descriptions of how the voices have ‘affected relationships’ and ‘what the voices mean’ indicate strongly positive voices and valences: for example, ‘not scary at all, reassuring’, ‘very kind and compassionate’, ‘intimately caring’, ‘total reassurance, help and understanding’, ‘total and overwhelming joy’, ‘deep compassion and love’ and ‘a sense of awe’. In fact, only one participant responded with a negative appraisal when asked what the voices have meant, saying that the voices have mostly caused ‘damage’. This same participant differed strikingly from the majority by also reporting multiple voices, non-spiritual voices, and abusive voices.

Box 2 **Participants’ reported significance of voices**

‘Everything’

‘Life enhancing. When my spiritual life feels dry I reflect on them. I feel protected’

‘Most precious and significant events of my life’

‘They have reconnected me with a childhood faith, lost years before, when I was left damaged and broken by life events’

‘Produced a sense of profound thankfulness’

‘The foundation of my life’

‘Increased knowledge, better understanding, increased self-confidence’

‘It has been wonderful, keeping me going in bad times’

‘Something that allowed my soul to grow and be nurtured and to come alive’

‘It means I am not alone’

‘They have been very, very precious. They sounded indescribably beautiful, and I have felt hopeful and glad…and reassured by them.’

‘[The experience of hearing this voice] gave me the strength to push on through my hurt to a better acceptance of myself’.

Table 5 provides a breakdown for voices according to the taxonomic categories summarised in table 1. The voices all communicated something (or in some way) deeply personal for the voice hearer. It is noteworthy that there were 15 instances of voices providing comfort. The second most prevalent category (14 cases) were those voice experiences that communicated a new sense of calling (usually an urging to join vocational Christian ministry) to the person hearing the voice.

**Table 5:** Taxonomic classification of voice-hearing experiences

Taxonomic category*	Example	n=58
Comfort	‘Do not be afraid’.	15 (26%)
Calling	‘Teach scripture’.	14 (24%)
Confirming/clarifying	‘You’re going to marry him, you know’.	8 (14%)
Conversion	‘Be baptised’.	5 (9%)
Communications	‘Walk away’.	5 (9%)
Crisis	‘Trust me!’	4 (7%)
Conversational	I chat to God everyday and that conversation often feels like dialogue.	4 (7%)
Companions	‘You couldn’t come to me, so I came to you’.	3 (5%)
Other	‘Shekinah’58	7 (12%)
Not enough information to classify		8 (14%)

*Some participants experienced voices in more than one category.

Twenty-one responses, overall, indicated that voices occurred sometimes or always during prayer. These were distributed across all eight taxonomic categories.

## Discussion

In our predominantly Christian sample, most spiritually significant voices began in adult life, and were more or less infrequent experiences. Half were experienced as originating internally and half externally; half were experienced as auditory and a quarter as more thought-like, with the rest being a mixture of auditory and thought-like. Only half were considered to be characterful. Almost 90% reported a voice that was divine and almost all heard the voice of a personal spiritual being in one form or another. Arguably, the spiritual identity of the voice is what makes the voice ‘spiritually significant’. However, when asked about this significance, respondents resorted to a wide vocabulary conveying the value and meaning of the experience in life context. Spiritually significant voices are something to be grateful for; they are ‘life enhancing’, ‘precious’, ‘wonderful’ sources of growth and nurture. They provide coping resources in times of adversity.

### Comparison with Woods *et al*

Our findings contrast with those of Woods *et al* in a number of ways. Unlike Woods *et al*, who observed that 81% of voice hearers heard multiple voices, we found the inverse—only 21% reported multiple voices. For those who did hear multiple voices, experiences were very diverse. For example, one subject reported hearing the voice of God, the voice of a spirit guide and the voice of a grandparent. Another subject heard the voice of God and the voice of the devil, on separate occasions.

Three quarters of our subjects did not report any psychiatric diagnosis, as compared with only 17% in their sample. Nearly all of our participants described overtly salutary or edifying outcomes from their voice-hearing experiences (panel 2), and comparatively few exhibited neutral emotional valence. This lack of negative or neutral emotional valence, and almost complete lack of abusive/violent voices, seems linked to the messages communicated by the spiritually significant voices.

Voices in our study were less likely to be characterful, but more likely to include commands. It is surprising that half of our subjects identify their voices as personal spiritual entities without characterful qualities. Generally, it would seem that spiritually significant voices are experienced as clearly individualised agents, capable of reasoned interaction. For 10%, there was even a conversational relationship with the voice. Given that the God of Christian scripture and tradition is clearly characterful, it was unexpected that our respondents would report that the voice of God (as they believed that they heard it) was not characterful. We can only speculate as to why this was, but perhaps ‘character or personality’ were perceived by the 50% of our respondents who gave a negative answer to this question as human qualities, inappropriate to the voice of God? In any event, 50% of our respondents did also affirm their voices as being characterful.

Voices giving commands are generally thought to be more common in those diagnosed with mental illness.33 There was a low rate of psychiatric diagnosis reported by our subjects as compared with those studied by Woods *et al*, and yet more subjects in our study gave examples of voices giving commands. We may speculate that the quality of these commands is likely to be different from that experienced by psychotic subjects. Luhrmann has suggested that ‘the commands feel less commanding’ for Christians hearing God’s voice.34 Data from our study do not enable us to confirm or refute that suggestion.

Only a quarter of our participants reported somatic phenomena associated with their voices, as compared with 66% of those studied by Woods *et al*. Of those subjects in our study who did recount bodily sensations, many used temperature terms such as ‘heat’ or ‘warmth’. Similarities in the language used to describe somatic sensations may reflect the cultural–linguistic overlap of the participants in the two studies (and in other major studies of voice hearing, most recruiting white English speakers from Western Europe and the USA). However, this common finding also highlights a phenomenological commonality in which, for some voice hearers, the auditory is inseparable from other sensory modalities.

As Woods *et al* intimate, additional research is required to investigate any potential link between bodily sensations and voice-hearing experiences that arose in physically traumatic childhood circumstances.35 The vast majority of our participants reported no bodily sensations and no violent voices, and this may well reflect completely different underlying causes. While Woods *et al* found nearly half of voice hearers first started hearing their voices in ‘negative’ or ‘traumatic’ circumstances, we again received insufficient data to make a direct comparison. A significant minority of our respondents (12%) first heard a spiritually significant voice in the midst of a personal or vocational shift of some sort—again, with no indication of whether this was deemed a positive or negative change.

Like Woods *et al*, approximately two-thirds of our participants identified as female. While evidence suggests that prevalence of hallucinatory experiences may be higher in women than in men,36 our findings also reflect religious demographics in Britain.

Like Woods *et al*, we found a 50/50 split between those who claimed to hear their voices as if originating externally (outside of their own heads/ears) and those reporting voices originating internally (within their own heads/ears). Unlike Woods *et al*, we found that over half of participants had only literally auditory voice-hearing experiences. Our subjects were far more likely to have either auditory or thought-like voices, rather than a mixture of the two. This may point to a significant phenomenological difference with a corresponding cognitive–neurological basis, or it may result more simply from our participants’ much lower number of voice-hearing experiences. With the majority reporting fewer than five individual instances of hearing a spiritually significant voice, they may be more capable of clearly recalling the details of these isolated events rather than blending frequent occurrences into a synthetic impression of the typical presentation.

All of our subjects, except one, were religious. Religious traditions foster environments and expectations conducive of having experiences of a spiritually significant agent. For example, one of our respondents wrote:

There was a mini revival going on with the work of the Holy Spirit being highlighted. I hadn't heard much about her/him before. Following Jesus was hard work. After asking the Holy Spirit to be in my life, very gently I knew there was a difference. I started getting the thoughts after that.

For non-religious subjects (perhaps including some of the 22% in the study by Woods *et al* who did not specify any religious beliefs), anomalous experiences may occur and then be simultaneously or retrospectively interpreted as involving a spiritual agent, but this requires a greater hermeneutical ‘leap’. Only 16% of the sample reported by Woods *et al* understood their voices to be supernatural or spiritual entities, and only 16% identified spiritual purpose in their voices. Other studies37 of spiritually significant voices indicate that, for those whose voice experiences occur outside of religious contexts or in the absence of any religious beliefs, spiritual interpretations/meanings may come years or even decades later – often after receiving new explanations from friends or relatives. Further research is required to compare spiritually significant voices arising within the traditions and experiences of a faith community, as compared with those who have no experience of religious faith, or who identify as ‘spiritual but not religious’.

### Taxonomy

Attempts to classify subtypes of AVHs have typically focused on such things as phenomenology, underlying mechanisms or diagnosis.38 Although some research has focused on voice hearers’ own interpretations of their experiences,39 taxonomies of voice-hearing experiences do not hitherto seem to have taken the content of what the voice says, or the context of voice hearing, very seriously. The taxonomy of spiritually significant voices proposed here clearly reflects predominantly Christian concerns, and we recognise that research undertaken in other faith communities may produce different results. However, most faith traditions seek to provide some kind of comfort to adherents, and it is interesting that this seemed to emerge as the most commonly experienced category of voice. If we also include voices heard in times of crisis and uncertainty, almost half of the respondents in our survey appear to have heard voices that were encouraging or affirming in life context.

It is perhaps not surprising that the second most commonly encountered category in the taxonomy was calling, and that most of these experiences were in the context of calling to ordained ministry within the Church. Many clergy read the *Church Times*, and this is clearly a selected sample by virtue of ascertainment through readership of that periodical. However, some of these stories are remarkable. Major changes in the lives of these Christians have been marked, if not actually caused, by the hearing of a spiritually significant voice, usually understood as the voice of God. Similarly, conversion experiences sometimes seem to be accompanied by the hearing of a voice.

We debated whether or not additional categories might be added to the taxonomy as originally developed by Cook (2020). Two participants’ experiences in the present study pointed to a potential ninth category of ‘canny’ (or prophetic) voices, which apparently predict future happenings. It is debatable as to whether or not they should be classified separately, except insofar as they appear eerily prescient. We do not know how many similar voices prove inaccurate in their predictions and thus are not reported as spiritually significant due to the benefit of hindsight.40 Three people reported voices that gave commands or ‘prompts’ which did not easily fit into other categories of the taxonomy and so were classified as ‘other’. However, commands and prompts also appeared in other categories in our taxonomy (eg, conversion and calling) and it has previously been suggested by McCarthy-Jones *et al* 41 that commands should be considered as a dimensional quality of voices that cuts across categories, rather than constituting a category per se.

This taxonomy remains provisional, but it points to the need for categories of spiritually significant voices that take seriously content and context, as well as phenomenology.

### Comparison with other studies

As with other studies,42 our findings indicate that Christians who hear a voice frequently interpret these experiences as messages from spiritual agents who address relatively mundane concerns or that provide comfort and reassurance.

A large portion of our participants were self-identified Anglicans, an unsurprising finding given our recruitment through an Anglican periodical.43 Dein and Littlewood44 speculated that Anglicans may be less open to concepts of external spiritual influence and more wedded to the notion of their own intentional agency than other groups of Christians. However, Dein and Cook45 provided qualitative accounts of eight members of an Anglican church in East London who did report experiences of communications from God. The experiences of these eight subjects were all thought like, with only one subject also reporting an auditory experience of a voice, whereas our study showed that over half of participants had only auditory voice-hearing experiences.

In the present study, our participants reported virtually no ability to predict, or to use their own agency to bring on, the voice(s), a finding shared by Dein and Littlewood’s Pentecostal sample. The inability to predict a voice may be related to a general lack of any accompanying bodily sensations. Dein and Littlewood similarly reported that their subjects only ‘sometimes’ experienced physical changes such as warmth or light-headedness. In contrast, in research on spiritualists reporting clairaudient experiences, both the ability to control/predict the onset of voices and bodily sensations are common.46

Approximately one-third of respondents in our survey gave some specific indication that their voices were heard in the context of prayer. Luhrmann *et al* 47 has suggested that it is ‘a proclivity for ‘absorption’’ that, in combination with learning, makes some people more likely to hear a voice in prayer than others. There is evidence suggesting that absorption may also be an important factor in the hearing of clairaudient voices among spiritualist mediums.48 However, many of our respondents appeared not to be in a state of absorption at the time of hearing their voices, or else were absorbed in other activities (eg, driving and studying) or were emotionally distressed.

In common with other studies of both Christian and spiritualist voices, our findings offer hints that the hearing of spiritually significant voices is accompanied by a process of discernment during and after the experience to determine whether or not the communication was of spiritual origin.49 For example, one subject reported ‘From initial suspicion and reluctance I have decided that it is God speaking’. Another respondent wrote:

I worry maybe its not God sometimes. What if it is all psychosomatic or something? But the more I grow as a Christian the less plausible that seems…… I am very aware of the dangers of claiming to have a direct line to God, so I need to be humble about all of this.

However, the significance of the voices for other participants was recalled unambiguously and with immense positive affect. For some, there appears to be no need for further discernment. For example, one subject reported that ‘I never doubt what I have received’. In respect of the identity of the voice, subjects report a strong sense or conviction of that identity (eg, as the voice of God), without feeling the need to provide evidence for that belief.

### Continuum or discontinuum?

While some studies have shown that non-clinical voice hearers begin hearing voices at an earlier age on average than clinical voice hearers,50 over half of our participants had their first voice experience as adults (21+ years of age). Over three-quarters had never received any mental health diagnosis at all. Consistent with Daalman *et al*,51 as well as with some other studies of spiritually significant voices,52 our participants had relatively few experiences of hearing a voice. For nearly one half, spiritual voices had only been experienced fewer than five times.

Our comparisons with previously published studies should not be taken to imply that we consider that Christian experiences of hearing spiritually significant voices are just another form of voice hearing. Neither do we necessarily reject this possibility. We have noted differences, as well as similarities in phenomenology. Our sample was not recruited on the basis of self-identification as a ‘voice hearer’, and we do not imagine that most of our respondents would see themselves as voice hearers, or their experiences as ‘voice hearing’.53 There are significant theological, sociological and other differences between being a voice-hearer as compared with being a Christian (or other person) who has heard a ‘spiritually significant voice’. Voice hearing is associated with decreased stigma where it is identified as the voice of God and has positive content.54

Ultimately, the commonalities in voice-hearing phenomenology across various populations, including the similarities of language used to describe accompanying bodily sensations, may still point to continuities between clinical and non-clinical, as well as spiritual and non-spiritual, voice-hearing experiences. On the other hand, differences in voice content, frequency, number, agency, as well as associated emotional valence, highlight the fault lines still separating these voice-hearing groups. These boundaries must be explored further by mental health researchers and need to be continually acknowledged by clinicians seeking to understand their patients in pluralistic, multicultural contexts.

### Limitations

Although our survey design and methodology were based on Woods *et al*,55 we narrowed the focus of our survey to gather demographic, contextual and phenomenological details specifically about spiritually significant voices. This meant recruiting from a smaller and more religious pool. Our efforts resulted in a largely British, Christian sample of individuals over 45 years of age. What we are able to say about spiritually significant voices is therefore limited to this demographic and is based on a small sample size.

Although designing a questionnaire with free-text responses allowed us to consider a greater diversity of experiences, with correspondingly greater nuance, it also meant receiving responses with wide variation in length and detail. Thus, we were unable to compare responses for some variables (eg, onset circumstances) and needed to introduce new categories (eg, ‘transitional’ circumstances of onset) to account for emerging patterns unique to our participants. This limited our capacity for direct comparison with previous studies. Similarly, while we intentionally desired a focused set of Christians who reported spiritually significant voices, the significant demographic homogeneity of our sample hinders wider cross-cultural comparison, even with other studies of religious voice-hearers.56

### Studying the phenomenology of religious experience

Despite its limitations, our methodology enabled us to explore the phenomenology of spiritually significant voices in such a way as to show that there are both continuities and discontinuities with other experiences of the hearing of voices. We found content and context to be significant, and neglected, aspects of these experiences and expect that there is still much to learn about them in future research. To this end, various disciplines have a part to play. For example, detailed ethnographic studies, following on from those already undertaken by Luhrmann, are needed in different religious and cultural contexts. Our findings also point to the particular value of involving the disciplines of theology and religious studies in these endeavours. Phenomenological studies of voice hearing within faith communities, often emphasising agency and control over voices, must consider how collectively held metaphysical beliefs delimit the nature and scope of those experiences. Spiritualists rarely experience unknown non-agentive voices because their monistic worldview virtually precludes communicative supernatural agents that were not once physical human individuals. In contrast, some of our Christian respondents differently nuanced their accounts of the hearing of the voice of Jesus, for example, as compared with voices of the Holy Spirit or ‘God’ (and none identified the voice explicitly as ‘God the Father’), reflecting Christian Trinitarian doctrine. For example, one respondent distinguished two ‘experiences of God’ from three ‘encounters with Jesus’, writing that ‘God was just a felt presence but Jesus has been visible’. In answer to the question ‘Does it feel as though the voice(s) that you hear have their own character or personality?’ this person wrote:

Definitely. There are different tones of voice. I have had Jesus being exasperated with me, gentle with me, laughing with me, challenging me, ordering me. God being both calmly implacable and gently encouraging.

Expectations of hearing a voice at conversion may be modelled on the biblical example of St Paul, and receiving auditory vocational callings may find their prototype in the biblical accounts of Moses or Isaiah. Religious voices are heard within the context of theological traditions, and those traditions hold important keys to interpretation.57

## Conclusions

The similarities between our sample and previously published studies of voice hearers and voice hearing are significant for a number of reasons. First, phenomenological similarities easily form the basis for misdiagnosis in the clinical setting. It is important that clinicians should be aware of such experiences as entirely normal phenomena in spiritual and religious context. The phenomenological differences highlighted in this study may make it appear that such mistakes would be unlikely (especially for the infrequent variety of spiritually significant voices), but in practice, there is reason to believe that they do occur. Even if they do not, the fear that a psychiatrist might misdiagnose such experiences is likely to make patients reticent in discussing them. Not only do psychiatrists need to be well informed about such matters, but also they must be *known* to be well informed.

Second, while the present sample of respondents found their experiences to be largely positive, spiritually significant voices may provide insights into possible positive coping mechanisms for Christians and others who hear distressing voices and thus, indirectly, new approaches to treatment. We can only speculate at this stage as to what such interventions might look like, but we may imagine that they will pay much more attention to the context, content and identity of the voices. These hitherto neglected features of the voice are central to the way in which the experience is interpreted and responded to. Thus, for example, contextualising demonic voices as an experience that Jesus had (as recorded in the Gospels), and not necessarily a sign of spiritual problems, might be very reassuring for a Christian distressed by the possibility that their voices indicate that they are separated from God.

Third, our findings draw attention to the need to attend to the possible spiritual/religious significance of all experiences of hearing a voice. While not everyone identifies as spiritual or religious, for those who do, their spirituality may well shape both the experience itself, and their interpretation of their experience, in significant ways. For some people, the voice is experienced, in the moment, as self-evidently spiritual. They may or may not later reflect on this and exercise a conscious process of discernment concerning the authenticity or meaning of the experience. Even for those who would not self-identify as spiritual/religious (who were clearly not included in our survey) we might imagine that similar processes occur. The meaning and significance of the voice may be understood as self-evidently not spiritual. However, on later reflection, this assessment may be revised in favour of spiritual or religious interpretations. While this process of reassessment will have positive consequences for some, it may prolong the distress for others.

## Data Availability

No data are available. Data for this study are not available, under terms of participant consent, for sharing outside the research team.

## Notes

1. Whitehead, Anne, and Angela Woods, eds (2016), *The Edinburgh Companion to the Critical Medical Humanities* (Edinburgh: Edinburgh University Press, 2016), 1.

2. Laurie Green (2009), *Let’s Do Theology: Resources For Contextual Theology*, Revised ed. (London: Continuum, 2009).

3. Even these exceptions may often not be seen as ‘medical humanities’ so much as interdisciplinary work on theology and health. See, for example, J Swinton (2001), *Spirituality and Mental Health Care* (London: Jessica Kingsley, 2001); Joel James Shuman and Keith G. Meador (2003), *Heal Thyself: Spirituality, Medicine, and the Distortion of Christianity* (Oxford: Oxford University Press, 2003); C. C. H Cook (2006), *Alcohol, Addiction and Christian Ethics* (Cambridge: Cambridge University Press, 2006); Christopher C H Cook (2004)., Christopher C. H Cook, 2013, *Spirituality, Theology & Mental Health* (London: SCM, 2013); Christopher C. H Cook (2018), *Hearing Voices, Demonic and Divine: Scientific and Theological Perspectives* (London: Routledge, 2018); Fraser Watts (2018), “Theology and Science of Mental Health and Well-Being,” *Zygon* 53, 2 (2018); John Swinton (2020), *Finding Jesus in the Storm: The Spiritual Lives of Christians with Mental Health Challenges* (Grand Rapids: Eerdmans, 2020).

4. Stephen Pattison (2007) "Absent Friends in Medical Humanities," *Med Humanit* 33, no. 2 (Dec 2007), https://doi.org/10.1136/jmh.2007.000263, https://www.ncbi.nlm.nih.gov/pubmed/23674423, has referred to theology and religious studies as being amongst the ‘absent friends’ of the medical humanities. The *Edinburgh Companion to the Critical Medical Humanities*, for example, includes no contribution from a theologian or anyone working in a department of religious studies.

5. See, for example, Angela Woods (2013), "The Voice Hearer," *Journal of Mental Health* 22, no. 3 (2013); Angela Woods et al. (2014), "Interdisciplinary Approaches to the Phenomenology of Auditory Verbal Hallucinations," *Schizophr Bull* 40, suppl (Jun 5 2014), https://doi.org/10.1093/schbul/sbu003, http://www.ncbi.nlm.nih.gov/pubmed/24903416; Patricia Waugh (2015), "The novelist as voice hearer," *The Lancet* 386, no. 10010 (2015), https://doi.org/10.1016/s0140-6736(15)01118-6; Corinne Saunders (2015), "Hearing Medieval Voices," *The Lancet* 386, no. 10009 (2015), https://doi.org/10.1016/s0140-6736(15)01061-2; Sam Wilkinson and Ben Alderson-Day (2015), "Voices and Thoughts in Psychosis: an Introduction," *Review of Philosophy and Psychology* (2015), https://doi.org/10.1007/s13164-015-0288-6; Charles Fernyhough (2016), *The Voices Within: The History and Science of How we Talk to Ourselves* (London: Profile, 2016); Cook, *Hearing Voices, Demonic and Divine: Scientific and Theological Perspectives*.

6. See, for example, Cook, *Hearing Voices, Demonic and Divine: Scientific and Theological Perspectives*; Christopher C. H Cook (2020), *Christians Hearing Voices: Affirming Experience and Finding Meaning* (London: Jessica Kingsley, 2020); Adam Powell and Peter Moseley (2020), "When Spirits Speak: Absorption, Attribution, and Identity Among Spiritualists Who Report 'Clairaudient' Voice Experiences," *Mental Health, Religion & Culture* (2020).

7. T. M. Luhrmann, *When God Talks Back* (New York: Knopf, 2012); Simon Dein and Roland Littlewood, "The Voice of God," *Anthropology & Medicine* 14, no. 2 (2007).

8. Robert N McCauley and George Graham (2020), *Hearing Voices and Other Matters of the Mind: What Mental Abnormalities Can Teach Us about Religions* (New York: Oxford University Press, 2020).

9. For example, Cook, *Christians Hearing Voices: Affirming Experience and Finding Meaning*.

10. See Saskia de Leede-Smith and Emma Barkus (2013) "AComprehensive Review of Auditory Verbal Hallucinations: Lifetime Prevalence, Correlates and Mechanisms in Healthy and Clinical Individuals." *Front Hum Neurosci* 7 (2013): 367. https://doi.org/10.3389/fnhum.2013.00367.https://www.ncbi.nlm.nih.gov/pubmed/23882203

11. Nev Jones, Timothy Kelly, and Mona Shattell (2016), "God in the Brain: Experiencing Psychosis in the Postsecular United States," *Transcult Psychiatry* 53, no. 4 (Aug 2016), https://doi.org/10.1177/1363461516660902, https://www.ncbi.nlm.nih.gov/pubmed/27495312; Adam Powell and Peter Moseley (2020), "When Spirits Speak: Absorption, Attribution, and Identity Among Spiritualists Who Report 'Clairaudient' Voice Experiences"; S. Jones, A. Guy, and J. A. Ormrod (2003), "A Q-methodological Study of Hearing Voices: A Preliminary Exploration of Voice Hearers’ Understanding of Their Experiences," *Psychology and Psychotherapy: Theory, Research and Practice* 76 (2003).

12. Cook, *Hearing Voices, Demonic and Divine: Scientific and Theological Perspectives*.

13. Cook, Christopher C. H (2012a), "Psychiatry in Scripture: Sacred Texts and Psychopathology," *The Psychiatrist* 36, no. 6 (2012).

14. J van Os et al. (2000), "Strauss (1969) Revisited: A Psychosis Continuum in the General Population?" *Schizophrenia Research* 45 (2000).

15. Flavie Waters and Charles Fernyhough (2019), "Auditory Hallucinations: Does a Continuum of Severity Entail Continuity in Mechanism?" *Schizophr Bull* (Feb 7 2019), https://doi.org/10.1093/schbul/sbz002, https://www.ncbi.nlm.nih.gov/pubmed/30753702.

16. T M Luhrmann (2017), "Diversity Within the Psychotic Continuum," *Schizophr Bull* 43, no. 1 (Jan 2017), https://doi.org/10.1093/schbul/sbw137, https://www.ncbi.nlm.nih.gov/pubmed/27872266.

17. Luhrmann, *When God Talks Back*; Dein and Littlewood, "The Voice of God"; Simon Dein and Roland Littlewood (2007), "God Put a Thought into My Mind: The Charismatic Christian Experience of Receiving Communications from God," *Mental Health, Religion & Culture* (2015), https://doi.org/10.1080/13674676.2014.1002761.

18. Woods, "The Voice Hearer."

19. Albert R Powers, Megan S Kelley, and Philip R Corlett (2017), "Varieties of Voice-Hearing: Psychics and the Psychosis Continuum," *Schizophr Bull* 43, no. 1 (Jan 2017), https://doi.org/10.1093/schbul/sbw133, https://www.ncbi.nlm.nih.gov/pubmed/28053132.

20. Powers, Kelley, and Corlett, "Varieties of Voice-Hearing: Psychics and the Psychosis Continuum"; Powell and Moseley, "When spirits speak: absorption, attribution, and identity among spiritualists who report 'clairaudient' voice experiences."

21. Powell and Moseley, "When spirits speak: absorption, attribution, and identity among spiritualists who report 'clairaudient' voice experiences"; Luhrmann, *When God Talks Back*.

22. Angela Woods et al. (2015)., "Experiences of Hearing Voices: Analysis of a Novel Phenomenological Survey," *Lancet Psychiatry* (2015), https://doi.org/10.1016/S2215-0366(15)00006-1.

23. S. Jones, A. Guy, and J. A. Ormrod (2003), "A Q-methodological Study of Hearing Voices: A Preliminary Exploration of Voice Hearers’ Understanding of Their Experiences."

24. Waugh, "The novelist as voice hearer."

25. Woods, "The Voice Hearer."

26. For further discussion, and a proposed definition, see Christopher C H Cook (2004), "Addiction and Spirituality," *Addiction* 99 (2004).

27. Cook (2017a), "Speak, Lord: Thy Servant Heareth," *Church Times*, no. 21 April (2017); Cook (2017), "Learning to Discern," *Church Times*, no. 28 April (2017); Cook (2017b), "Voices Immanent and Transcendent," *Church Times*, no. 5 May (2017).

28. Cook, *Christians Hearing Voices: Affirming Experience and Finding Meaning*.

29. Cook, *Christians Hearing Voices: Affirming Experience and Finding Meaning*. See especially the discussion on pp24-25.

30. Cook, *Christians Hearing Voices: Affirming Experience and Finding Meaning*, 129-42.

31. Luhrmann, T. M (2012b) *When God Talks Back*. (New York, Knopf, 2012)

32. In a study of writers’ experiences of hearing the voices of their characters, 33% distinguished these voices from their own inner speech (John Foxwell et al. (2020), "'I've learned I need to treat my characters like people': Varieties of agency and interaction in Writers' experiences of their Characters' Voices," *Consciousness and Cognition*, 79 (Mar 2020), https://doi.org/10.1016/j.concog.2020.102901, https://www.ncbi.nlm.nih.gov/pubmed/32113151.). The distinction here is more about multiple voices distinguished both from inner speech and from one another. Whether or not these different voices might be considered similar to the different characters in a writer’s mind, or perhaps more qualitatively different from one another in some way, remains to be explored in future research.

33. Tanya Marie Luhrmann et al. (2019)., "Beyond Trauma: A Multiple Pathways Approach to Auditory Hallucinations in Clinical and Nonclinical Populations," *Schizophr Bull* 45, no. 45 Suppl 1 (Feb 1 2019), https://doi.org/10.1093/schbul/sby110, https://www.ncbi.nlm.nih.gov/pubmed/30715545.

34. Luhrmann, "Diversity Within the Psychotic Continuum," 28.

35. See also research suggesting that negative voice content partially mediates the relationship between childhood trauma and distress: Cherise Rosen et al. (2018)., "Negative Voice-Content as a Full Mediator of a Relation Between Childhood Adversity and Distress Ensuing from Hearing Voices," *Schizophrenia Research* 199 (Sep 2018), https://doi.org/10.1016/j.schres.2018.03.030, https://www.ncbi.nlm.nih.gov/pubmed/29580740.

36. John J McGrath et al. (2015)., "Psychotic Experiences in the General Population: A Cross-National Analysis Based on 31261 Respondents From 18 Countries," *JAMA Psychiatry* 72, no. 7 (Jul 1 2015), https://doi.org/10.1001/jamapsychiatry.2015.0575, http://www.ncbi.nlm.nih.gov/pubmed/26018466.

37. Powell and Moseley, "When spirits speak: absorption, attribution, and identity among spiritualists who report 'clairaudient' voice experiences."

38. Simon McCarthy-Jones et al. (2014)., "Better Than Mermaids and Stray Dogs? Subtyping Auditory Verbal Hallucinations and Its Implications for Research and Practice," *Schizophr Bull* 40 Suppl 4 (Jul 2014), https://doi.org/10.1093/schbul/sbu018, https://www.ncbi.nlm.nih.gov/pubmed/24936087.

39. See, for example, S. Jones, A. Guy, and J. A. Ormrod (2003), "A Q-methodological Study of Hearing Voices: a Preliminary Exploration of Voice Hearers’ Understanding of Their Experiences."

40. One such example came to the attention of one of us (CCHC) in the course of collecting accounts published elsewhere (Cook, *Christians Hearing Voices: Affirming Experience and Finding Meaning*.). Unfortunately, the person concerned was not willing to allow publication of the story, and clearly they did not contribute to the present survey or—if they did—they did not include reference in their responses to the experience in question.

41. McCarthy-Jones et al., "Better Than Mermaids and Stray Dogs? Subtyping Auditory Verbal Hallucinations and Its Implications for Research and Practice."

42. Cook, *Christians Hearing Voices: Affirming Experience and Finding Meaning*; Dein and Littlewood, "The Voice of God."; Dein and Cook, "God Put a Thought into My Mind: the Charismatic Christian Experience of Receiving Communications from God."

43. In the British Social Attitudes Survey, among the 38% of the British population that claims to ‘belong’ to the Christian religion, nearly a third (the largest subtype) specifically identify themselves as ‘Church of England/Anglican’ (see J Curtice et al. (2019)., *British Social Attitudes: The 36th Report* (London: The National Centre for Social Research, 2019).)

44. Dein and Littlewood, "The Voice of God."

45. Simon Dein and Christopher C H Cook (2015), "God Put a Thought into My Mind: the Charismatic Christian Experience of Receiving Communications from God."

46. However, in contrast to the study by Woods et al, they do not correlate with abusive or violent voices. See Powell and Moseley, "When spirits speak: absorption, attribution, and identity among spiritualists who report 'clairaudient' voice experiences."; Greg Taylor and Craig Murray (2012), "A qualitative investigation into non-clinical voice hearing: what factors may protect against distress?" *Mental Health, Religion & Culture* 15, no. 4 (2012), https://doi.org/10.1080/13674676.2011.577411.

47. T. M. Luhrmann, Howard Nusbaum, and Ronald Thisted (2010), "The Absorption Hypothesis: Learning to Hear God in Evangelical Christianity," *American Anthropologist* 112, no. 1 (2010), https://doi.org/10.1111/j.1548-1433.2009.01197.x.

48. Powell and Moseley, "When Spirits Speak: Absorption, Attribution, and Identity Among Spiritualists Who Report 'Clairaudient' Voice Experiences."

49. Dein and Littlewood, "The Voice of God."; Dein and Cook, "God Put a Thought into My Mind: the Charismatic Christian Experience of Receiving Communications from God"; Powell and Moseley, "When Spirits Speak: Absorption, Attribution, and Identity Among Spiritualists Who Report 'Clairaudient' Voice Experiences."

50. Kirstin Daalman et al. (2011)., "The Same or Different? a Phenomenological Comparison of Auditory Verbal Hallucinations in Healthy and Psychotic Individuals," *J Clin Psychiatry* 72, no. 3 (Mar 2011), https://doi.org/10.4088/JCP.09m05797yel, http://www.ncbi.nlm.nih.gov/pubmed/21450152; Albert R Powers, Megan S Kelley, and Philip R Corlett (2017), "Varieties of Voice-Hearing: Psychics and the Psychosis Continuum."

51. Daalman et al., "The same or different? A phenomenological comparison of auditory verbal hallucinations in healthy and psychotic individuals." The non-psychotic subjects studied by Daalman et al, although hearing voices less frequently than the psychotic subjects, were hearing voices at least once per month, a significantly higher frequency than for the majority of our subjects.

52. Dein and Littlewood, "The Voice of God."

53. Woods, "The Voice Hearer."

54. Peter L. Phalen et al. (2018)., "The Stigma of Voice-Hearing Experiences: Religiousness and Voice-Hearing Contents Matter," *Stigma and Health* 3, no. 1 (2018), https://doi.org/10.1037/sah0000075.

55. Woods et al., "Experiences of Hearing Voices: Analysis of a Novel Phenomenological Survey."

56. Luhrmann et al., "Beyond Trauma: A Multiple Pathways Approach to Auditory Hallucinations in Clinical and Nonclinical Populations."

57. Cook, *Hearing Voices, Demonic and Divine: Scientific and Theological Perspectives*.

58. A Hebrew word referring to the glory of the divine presence.
